# Supplementary material for: Striatal Isolated from Cyathus striatus Extracts Induces Apoptosis in Human Pancreatic Cancer Cells
Source: Molecules. 2022 Apr 24;27(9):2746. doi: 10.3390/molecules27092746 (PMC9103111; doi:10.3390/molecules27092746)
Supplement: Supplementary file 1 [file molecules-27-02746-s001.zip › molecules-1662286-supplementary.pdf]

Supplementary Materials

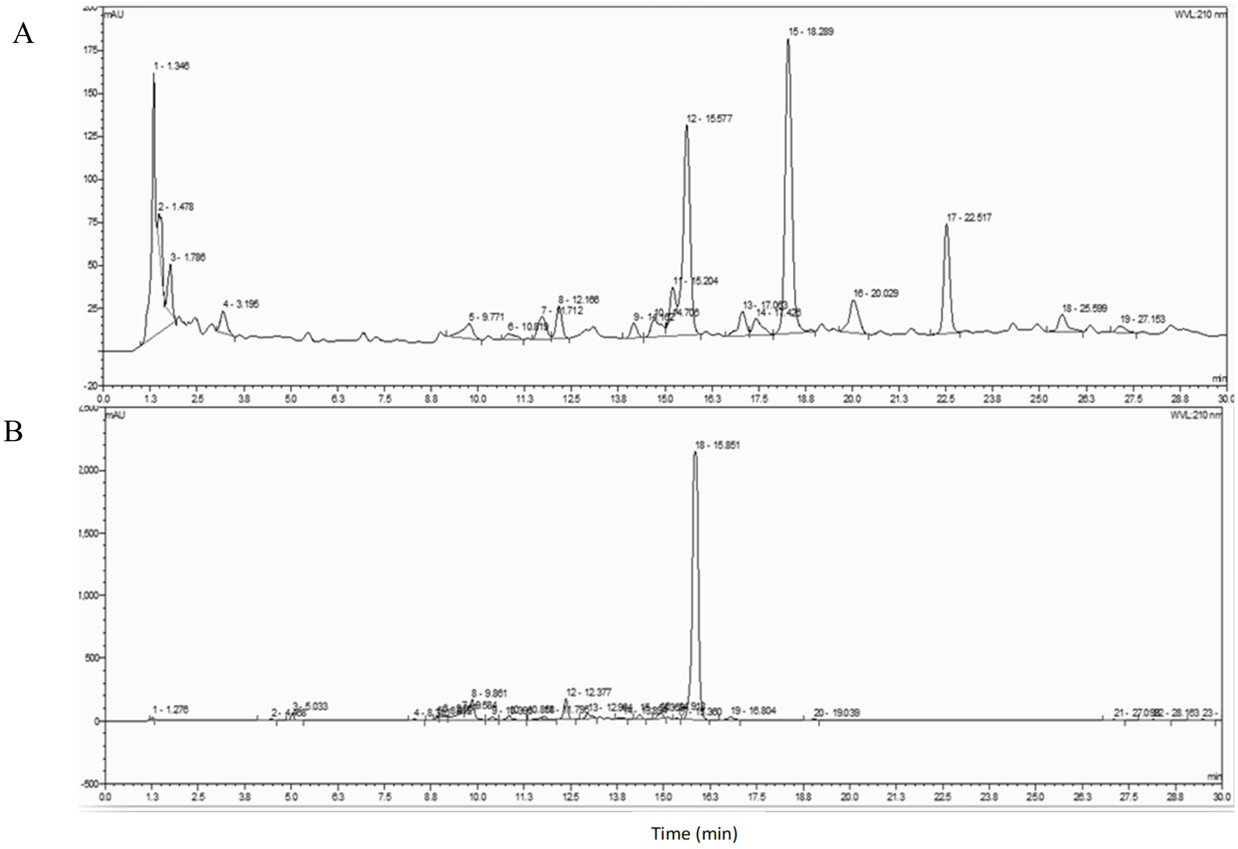

Figure S1. (A) HPLC chromatogram of the mushroom ethyl acetate crude extract (2 mg/ml). (B) The purified active compound (2 mg/ml) obtained at 210 nm using photodiode array detector.

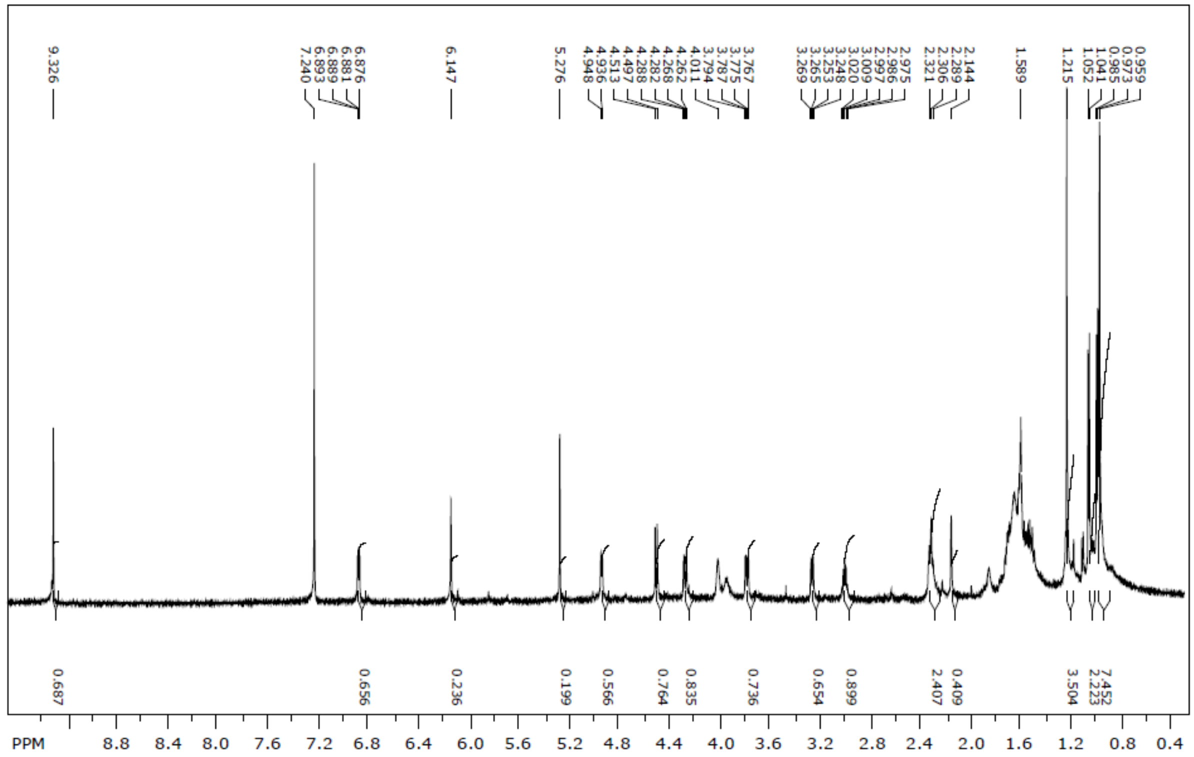

file: ...\NMR\data\Migal\nmr\Fadi600\23\fid expt: <zg>
transmitter freq.: 600.553003 MHz
time domain size: 131072 points
width: 15000.00 Hz = 24.9770 ppm = 0.114441 Hz/pt
number of scans: 8

freq. of 0 ppm: 600.550027 MHz
processed size: 262144 complex points
LB: 0.000 GF: 0.0000
Hz/cm: 226.034 ppm/cm: 0.37638

Figure S2: ^{1}H-NMR for the compound appeared at 16 min. in the HPLC chromatogram.

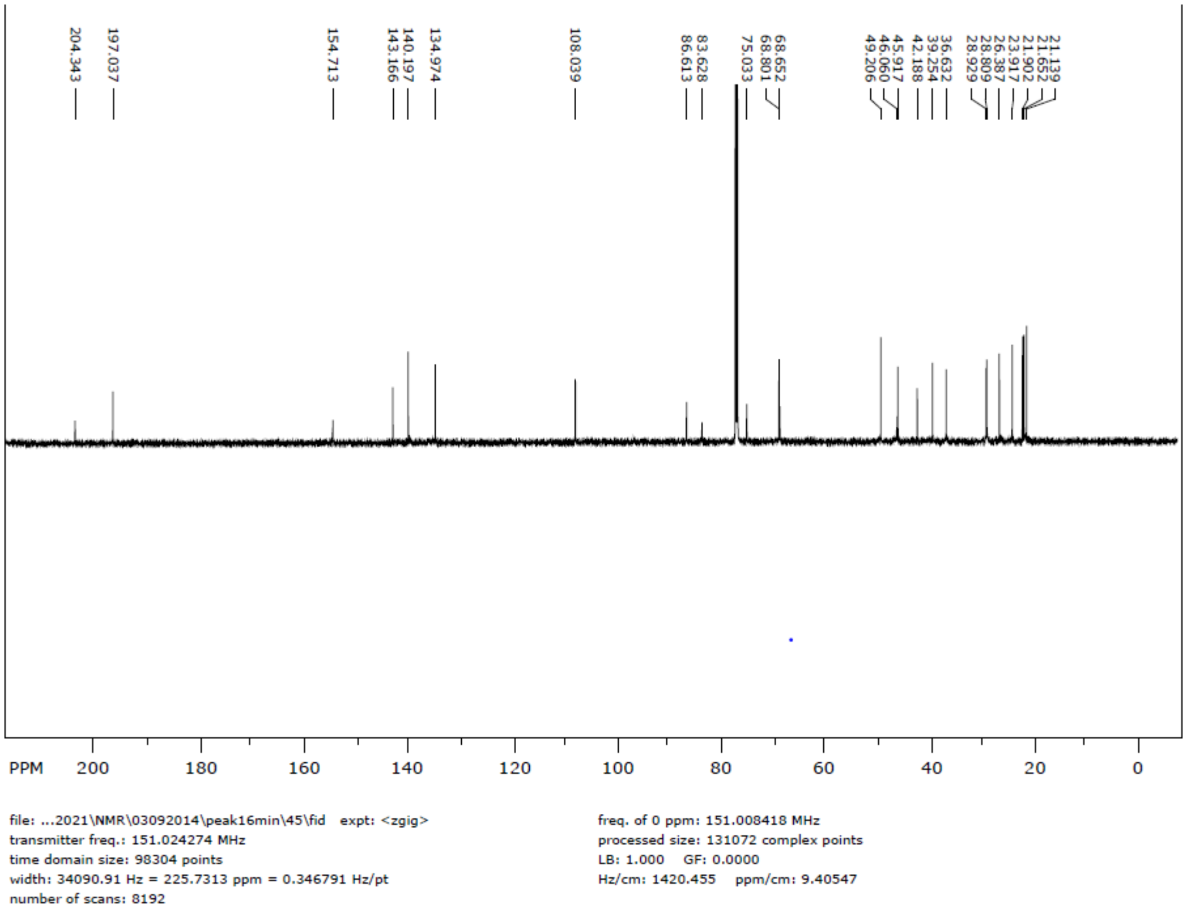

Figure S3: ^{13}C-NMR (zgig) for the compound appeared at 16 min. in the HPLC chromatogram.

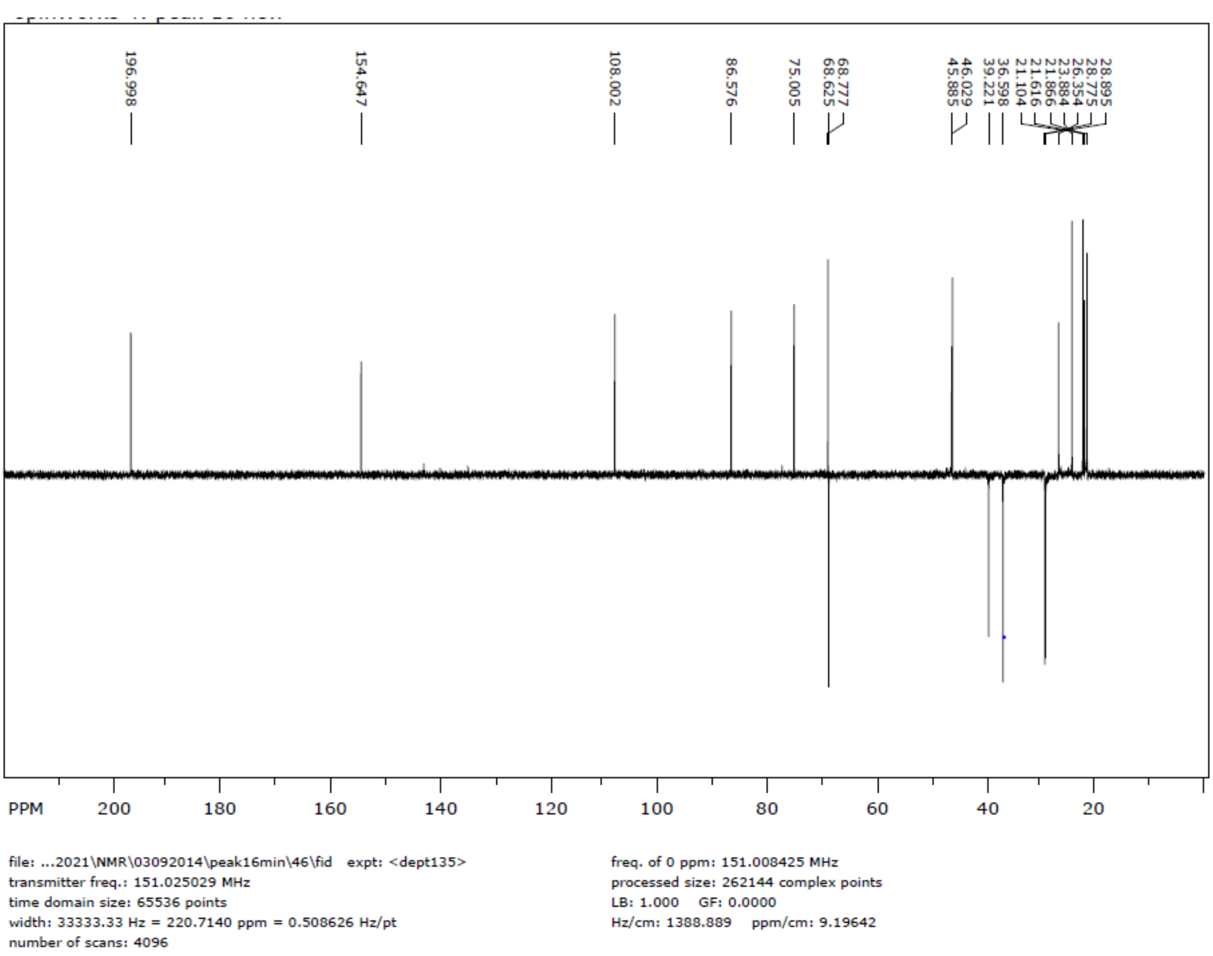

Figure S4: ^{13}C-NMR (dept135) for the compound appeared at 16 min. in the HPLC chromatogram.

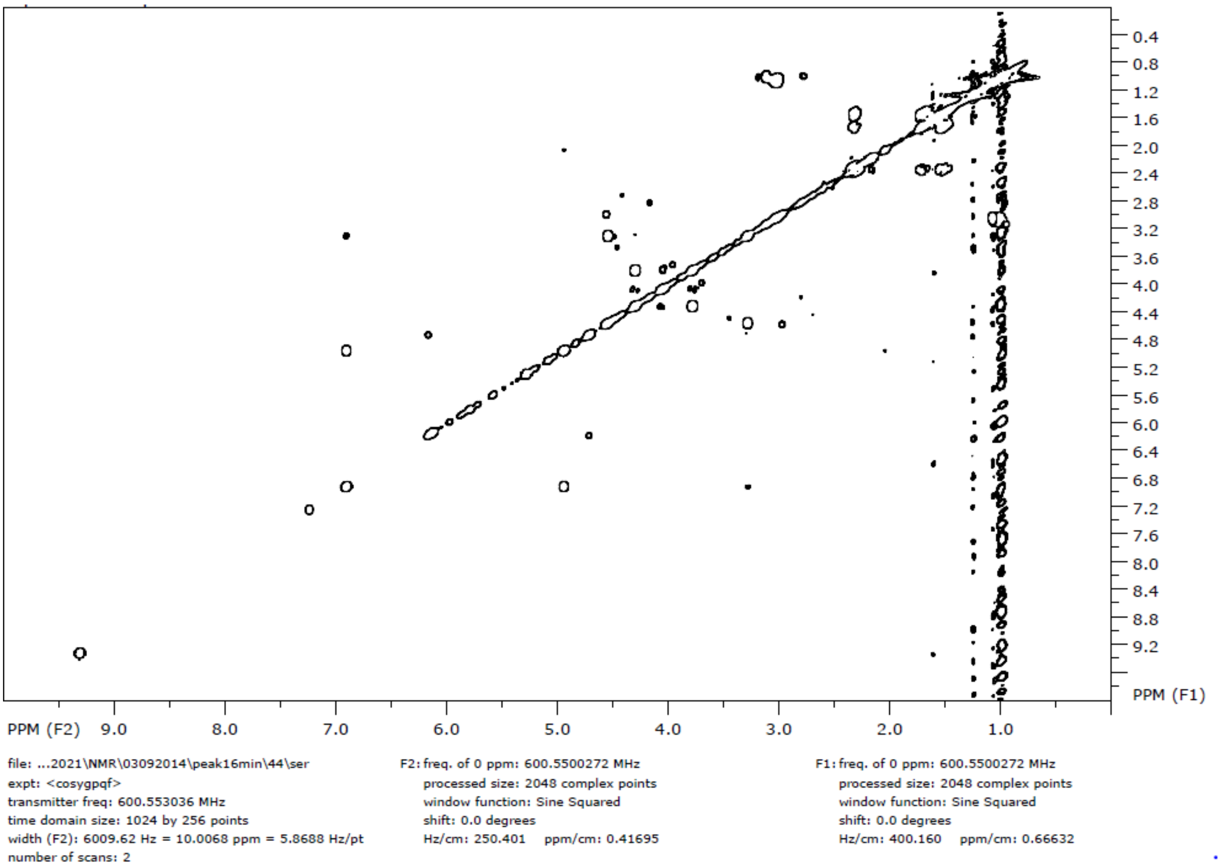

Figure S5: Two dimensional ^{1}H-^{1}H NMR (cosygpqf) for the compound appeared at 16 min. in the HPLC chromatogram.

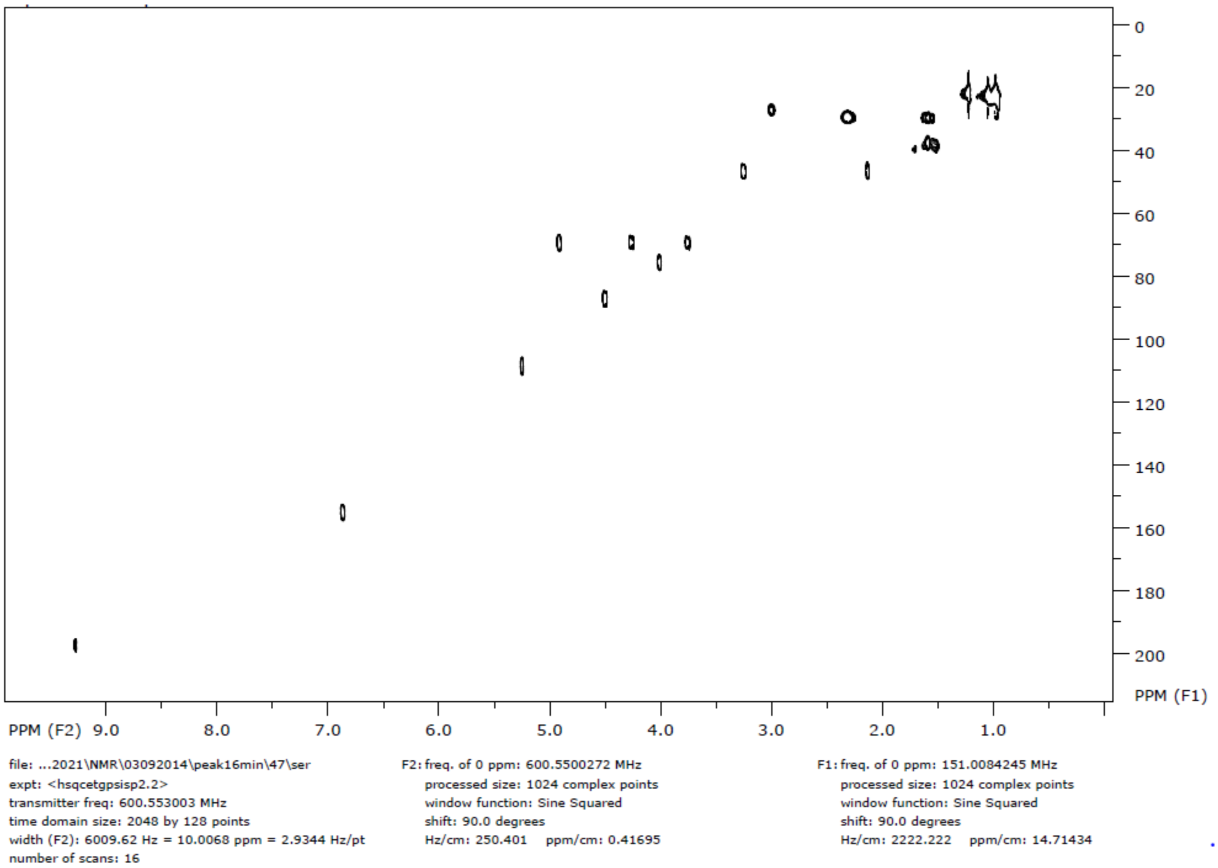

Figure S6: Two dimensional ^{1}H-^{13}C NMR (HSQC) for the compound appeared at 16 min. in the HPLC chromatogram.

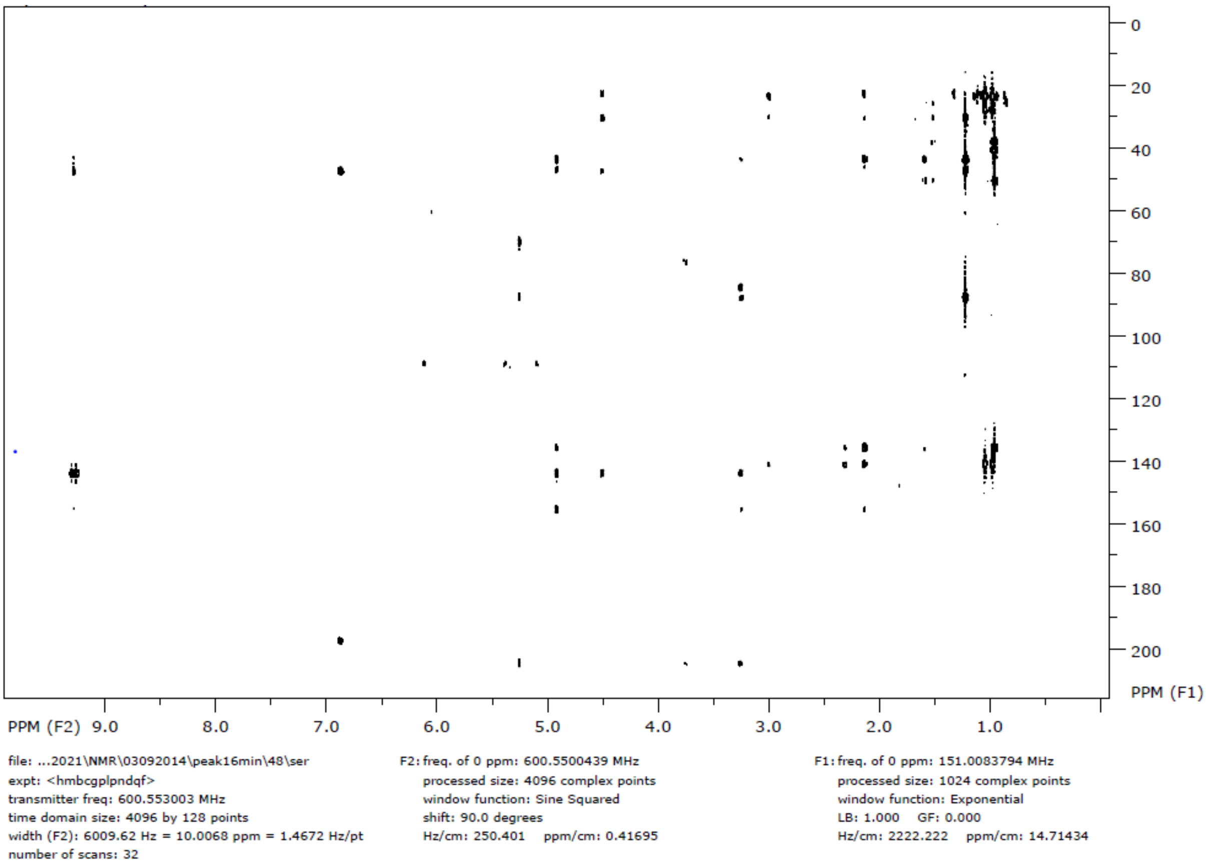

Figure S7: Two dimensional ^{1}H-^{13}C NMR (HMBC) for the compound appeared at 16 min. in the HPLC chromatogram.
